# Cytosine-5 methylation-directed construction of a Au nanoparticle-based nanosensor for simultaneous detection of multiple DNA methyltransferases at the single-molecule level†

**DOI:** 10.1039/d0sc03240a

**Published:** 2020-08-25

**Authors:** Li-Juan Wang, Xiao Han, Jian-Ge Qiu, BingHua Jiang, Chun-Yang Zhang

**Affiliations:** College of Chemistry, Chemical Engineering and Materials Science, Collaborative Innovation Center of Functionalized Probes for Chemical Imaging in Universities of Shandong, Key Laboratory of Molecular and Nano Probes, Ministry of Education, Shandong Provincial Key Laboratory of Clean Production of Fine Chemicals, Shandong Normal University Jinan 250014 China cyzhang@sdnu.edu.cn; Academy of Medical Sciences, Zhengzhou University Zhengzhou 450000 China bhjiang@zzu.edu.cn

## Abstract

DNA methylation at cytosine/guanine dinucleotide islands (CpGIs) is the most prominent epigenetic modification in prokaryotic and eukaryotic genomes. DNA methyltransferases (MTases) are responsible for genomic methylation, and their aberrant activities are closely associated with various diseases including cancers. However, the specific and sensitive detection of multiple DNA MTases has remained a great challenge due to the specificity of the methylase substrate and the rareness of methylation-sensitive restriction endonuclease species. Here, we demonstrate for the first time the cytosine-5 methylation-directed construction of a Au nanoparticle (AuNP)-based nanosensor for simultaneous detection of multiple DNA MTases at the single-molecule level. We used the methyl-directed endonuclease GlaI to cleave the site-specific 5-methylcytosine (5-mC). In the presence of CpG and GpC MTases (*i.e.*, M.SssI and M.CviPI), their hairpin substrates are methylated at cytosine-5 to form the catalytic substrates for GlaI, respectively, followed by simultaneous cleavage by GlaI to yield two capture probes. These two capture probes can hybridize with the Cy5/Cy3–signal probes which are assembled on the AuNPs, respectively, to form the double-stranded DNAs (dsDNAs). Each dsDNA with a guanine ribonucleotide can act as the catalytic substrate for ribonuclease (RNase HII), inducing recycling cleavage of signal probes to liberate large numbers of Cy5 and Cy3 molecules from the AuNPs. The released Cy5 and Cy3 molecules can be simply quantified by total internal reflection fluorescence (TIRF)-based single-molecule imaging for simultaneous measurement of M.SssI and M.CviPI MTase activities. This method exhibits good specificity and high sensitivity with a detection limit of 2.01 × 10^−3^ U mL^−1^ for M.SssI MTase and 3.39 × 10^−3^ U mL^−1^ for M.CviPI MTase, and it can be further applied for discriminating different kinds of DNA MTases, screening potential inhibitors, and measuring DNA MTase activities in human serum and cell lysate samples, holding great potential in biomedical research, clinical diagnosis, drug discovery and cancer therapeutics.

## Abstract 2

Cytosine-5 methylation-directed construction of Au nanoparticle-based nanosensors enables specific and sensitive detection of multiple DNA methyltransferases.

## Graphical abstract

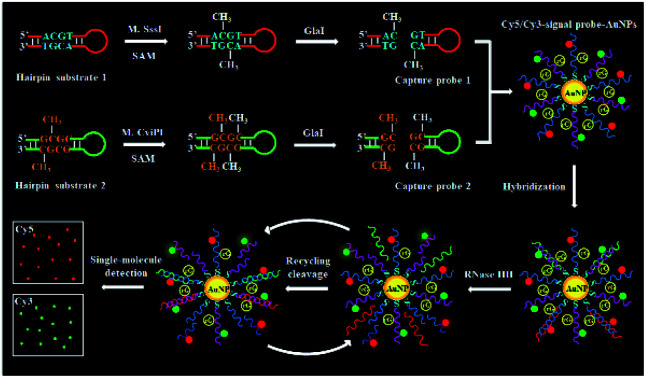

## Introduction

DNA methylation, frequently occurring at the carbon 5 (C^5^) position of cytosine to give 5-methylcytosine (5-mC) in cytosine/guanine dinucleotide islands (CpGIs), is the most prominent form of characterized epigenetic modifications in both prokaryotes and eukaryotes.^1,2^ Each CpGI has several tens to hundreds of CpG repetitions to constitute the main gene promoter regions,^3^ and hypermethylation of CpGI may disturb the critical gene silencing mechanisms, causing deregulation of diverse physiological functions.^4–6^ To maintain cellular DNA methylation patterns, DNA methyltransferase (MTase), a superfamily of cytosine methylases, can specifically recognize the palindromic sequences (*i.e.*, 5′-C-G-3′ or 5′-G-C-3′) and catalyze the transfer of a methyl group from *S*-adenosyl-l-methionine (SAM) to the cytosine in genomic DNA, balancing the methylation and demethylation status.^1,7,8^ Aberrant expression of DNA MTases may cause the malfunction of DNA methylation modification, resulting in various diseases^9–11^ and cancers.^12–14^ Therefore, DNA MTases may function as novel biomarkers for disease onset and important targets for cancer therapy, and the development of an efficient DNA MTase assay may facilitate the advance of methylase-based therapeutic strategies, clinical diagnosis, and drug discovery.

The conventional methods include radioactive labeling-based gel electrophoresis,^15^ enzyme-linked immunoassay,^16^ and high-performance liquid chromatography,^17^ but they suffer from intrinsic drawbacks of hazardous radiation labeling,^15^ costly protein antibodies,^16^ tedious sample preparation,^17^ low detection sensitivity,^15,16^ and time- and labor-consuming experimental procedures.^15–17^ Alternatively, some new approaches including colorimetric,^18,19^ luminescence,^20^ electrochemical,^21–24^ and fluorescence assays^25,26^ have been developed to overcome the limitations. For example, the colorimetric assay takes advantage of methylation-responsive DNA–gold nanoparticle (AuNP) assembly^18^ and terminal protection-directed DNA–AuNP diffusion^19^ for visualized detection of Dam MTase and DNA cytosine-5 methyltransferase (Dnmt 1), but it exhibits relatively poor sensitivity. The luminescence assay combines methylation-resistant cleavage with *in vitro* luciferase protein expression for the Dam MTase assay,^20^ but it involves cumbersome probe preparation, complex luciferase expression, and long assay time. In addition, a series of electrochemical^21–24^ and fluorescence assays^25,26^ take advantage of novel nanomaterials and methylation-sensitive restriction endonucleases (MSREs) for the quantification of Dam,^21,24,25^ M.SssI,^22^ Dnmt 1 (ref. 23) and HaeIII MTases,^26^ but they involve complicated nanomaterial synthesis,^21,23–26^ tedious electrode modification,^21–24^ and time-consuming protocols.^21–24,26^ To improve detection sensitivity, some nucleic acid amplification approaches have been introduced for the DNA MTase assay, including strand displacement amplification (SDA),^27^ rolling circle amplification (RCA),^28^ exponential isothermal amplification (EXPAR),^29^ and exonuclease-/endonuclease-assisted signal amplification (EASA).^30^ Despite the improved sensitivity, these assays are compromised by intricate multistep reactions,^28,29^ complicated circle-template synthesis,^28^ multiple primers and special polymerases,^27–29^ and usually suffer from high background.^27–30^ Notably, all the DNA MSREs involved in the DNA MTase assays are unmethylation-dependent,^18,21–29^ and they may cause false positivity even in the presence of trivial unmethylated DNAs uncleaved. Due to the specificity of methylase substrates and the rareness of MSRE species, all the reported methods enable the detection of only a single type of DNA MTase. Therefore, the development of a simple, accurate, and sensitive method for simultaneous detection of multiple DNA MTases remains a great challenge.

Unlike MSREs that identify and cleave unmethylated DNAs, GlaI is a newly discovered methyl-directed DNA restriction endonuclease with good specificity toward 5-mC and high activity toward various catalytic substrates, and it can recognize and cleave the site-specific methylated cytosines, with unmethylated cytosines remaining intact.^31,32^ Generally, MSRE-based DNA MTase assays are based on detection of uncleaved methylated DNAs with the assumption that unmethylated DNAs are completely cleaved.^33^ In fact, methylated DNAs actually account for a very small percentage of whole genomic DNA,^34^ and consequently even a trivial portion of incomplete cleaved unmethylated DNAs may cause significant interference. Because of the good specificity of GlaI toward methylated DNAs and high activity toward various catalytic substrates, the GlaI-cleaved fragments of methylated DNA can be specifically and sensitively discriminated from unmethylated DNAs, and this principle can be applied for simultaneous quantification of multiple low-abundance DNA MTases. Recently, owing to the remarkable advantages of ultrahigh sensitivity, high signal-to-noise ratio, and low sample consumption, single-molecule detection has become a powerful analytical technique in the fields of physics, chemistry, and biology,^35^ and has been successfully applied for sensitive detection of DNAs,^36^ miRNAs,^37^ enzymes,^38–40^ and epigenetic modifications.^41^ In this research, we take advantage of the unique features of GlaI and the intrinsic superiorities of the single-molecule detection technique, and demonstrate for the first time the cytosine-5 methylation-directed construction of a AuNP-based nanosensor for simultaneous detection of multiple DNA MTases at the single-molecule level. The presence of M.SssI and M.CviPI MTase may induce the methylation of cytosine residues in hairpin substrates (HS1 and HS2) to form the catalytic substrates (*i.e.*, 5′-A-mC-G-T-3′ and 5′-G-mC-G-mC-3′) of GlaI. GlaI may recognize the 5-mC sites and cut them to yield capture probes (CP1 and CP2). The capture probes can hybridize with signal probes (SP1 and SP2) functionalized on AuNPs to initiate ribonuclease (RNase HII)-mediated recycling cleavage of signal probes for the liberation of fluorescent molecules (Cy5 and Cy3, two cyanine dyes that are the most frequently used fluorescent dyes for biomolecule labeling).^42,43^ The M.SssI and M.CviPI MTase activities can be simultaneously detected by simply monitoring the Cy5 and Cy3 molecules. This method is highly specific and sensitive with a detection limit of 2.01 × 10^−3^ U mL^−1^ for M.SssI and 3.39 × 10^−3^ U mL^−1^ for M.CviPI MTases. Moreover, it can be used to discriminate different DNA MTases, screen DNA MTase inhibitors, and measure DNA MTase activities in human serum and cell lysate samples.

## Results and discussion

**Scheme 1:**
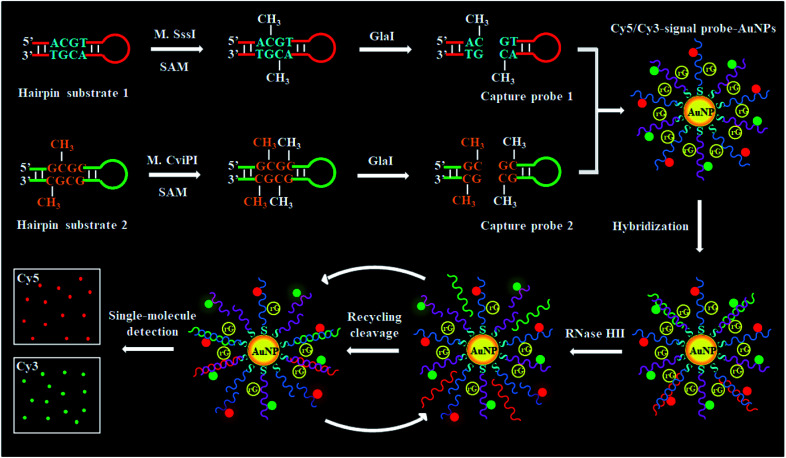
Schematic illustration of cytosine-5 methylation-directed construction of a AuNP-based nanosensor for simultaneous detection of multiple DNA MTases (*i.e.*, M.SssI and M.CviPI MTases) at the single-molecule level. This assay consists of three steps: (1) DNA MTase-catalyzed cytosine-5 methylation for inducing the cleavage of hairpin substrates by GlaI, (2) RNase HII-mediated recycling cleavage of signal probes for liberating Cy5 and Cy3 molecules from the AuNP nanostructures, and (3) simultaneous detection of Cy5 and Cy3 molecules at the single-molecule level.

To demonstrate the simultaneous detection of multiple DNA MTases, CpG MTase (M.SssI) and GpC MTase (M.CviPI) are utilized as the model enzymes. M.SssI and M.CviPI can methylate all cytosines at the C^5^ position within the palindromic sequences 5′-C-G-3′ and 5′-G-C-3′, respectively, in higher eukaryotes.^44^ This assay consists of three consecutive steps: (1) DNA MTase-catalyzed cytosine-5 methylation for inducing the cleavage of hairpin substrates by GlaI, (2) RNase HII-mediated recycling cleavage of signal probes for liberating Cy5 and Cy3 molecules from the AuNP nanostructures, and (3) simultaneous detection of Cy5 and Cy3 molecules at the single-molecule level. As illustrated in Scheme 1, we design two hairpin substrates (HS1 and HS2) with a loop and a stem, respectively. In both HS1 and HS2 (Scheme 1, red and green color), their stems contain the dinucleotide sequences 5′-A-C-G-T-3'/3′-T-G-C-A-5′ and 5′-G-mC-G-C-3'/3′-mC-G-C-G-5′, which may function as the catalytic substrates of DNA MTases (*i.e.*, M.SssI and M.CviPI), respectively, and the recognition sequences of DNA endonuclease (*i.e.*, GlaI) upon methylation reactions.^31^ The loops are partly complementary to the signal probes (*i.e.*, SP1 and SP2), respectively, whose hybridization may initiate the RNase HII-mediated recycling liberation of Cy5 and Cy3 molecules from the Cy5/Cy3–signal probe–AuNP nanostructures. The signal probes (*i.e.*, SP1 and SP2) (Scheme 1, blue and pink color) with sulfhydryl (SH) group modification at 3′ ends are labeled with the fluorophores (Cy5 and Cy3) on thymines positioned 5 and 4 bases and the guanine (G) ribonucleotides positioned 6 bases downstream of the 5′ ends in SP1 and SP2, respectively. The signal probes are functionalized on the surface of AuNPs *via* the S–Au covalent bonds to form the Cy5/Cy3–signal probe–AuNP nanostructures, in which the fluorescence of both Cy5 and Cy3 is efficiently quenched due to the strong quenching capability of AuNPs.^45,46^ In the presence of M.SssI MTase, the methyl group from *S*-adenosylmethionine (SAM) can be transferred to the cytosine-5 in the palindromic sequence 5′-A-C-G-T-3′ of HS1, forming the catalytic substrate 5′-A-mC-G-T-3′ for GlaI. Subsequently, the methylated HS1 is cleaved at the site 5-mC by GlaI, yielding a 25 nt capture probe 1 (CP1) (Scheme 1, red color). Similarly, in the presence of M.CviPI MTase, HS2 can be methylated to form another catalytic substrate 5′-G-mC-G-mC-3′ for GlaI, and the methylated HS2 is successively cut in the middle of dinucleotide sequences (*i.e.*, 5′-G-mC-G-mC-3'/3′-mC-G-mC-G-5′) to yield a 23 nt capture probe 2 (CP2) (Scheme 1, green color). With the addition of SP1/SP2-functionalized AuNPs into the reaction system, the resultant CP1 and CP2 can hybridize with SP1 and SP2, respectively, to form the double-stranded DNA (dsDNA) duplexes with each containing a G ribonucleotide. The dsDNA duplexes with G ribonucleotides can act as the catalytic substrates for the ribonuclease (RNase HII, an endoribonuclease that can specifically excise any single ribonucleotides misincorporated in genomic DNA by a one-step hydrolysis reaction),^47^ inducing the RNase HII-mediated recycling cleavage of signal probes and simultaneously liberating large numbers of Cy5 and Cy3 molecules from the AuNP nanostructures. The M.SssI and M.CviPI MTase activities can be simultaneously quantified by simply counting the Cy5 and Cy3 molecules liberated into the solution. In contrast, in the absence of M.SssI and M.CviPI MTases, HS1 and HS2 cannot be methylated and cleaved by GlaI, and neither CP1 nor CP2 is generated. Consequently, neither the cleavage of SP1 and SP2 nor the liberation of Cy5 and Cy3 molecules occurs, and thus neither the Cy5 nor Cy3 signal can be detected. Due to the ultrahigh specificity of GlaI toward 5-mC, the high specificity and efficiency of RNase HII-catalyzed single-ribonucleotide excision-mediated recycling amplification, and the high signal-to-noise ratio of single-molecule detection, the proposed method can simultaneously detect multiple DNA MTase activities with high accuracy and sensitivity.

**Fig. 1:**
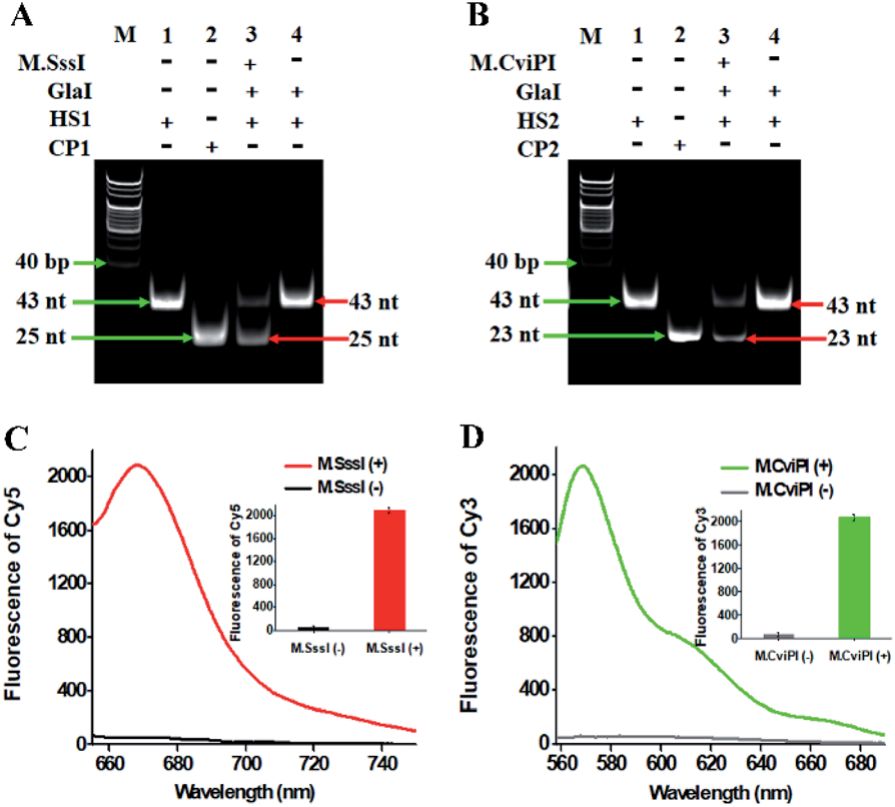
(A) Nondenaturating PAGE analysis of the cytosine-5 methylation catalyzed by M.SssI and the successive cleavage of HS1 by GlaI. Lane M, the DNA ladder marker; lane 1, the synthesized HS1; lane 2, the synthesized CP1; lane 3, in the presence of M.SssI + GlaI + HS1; lane 4, in the presence of GlaI + HS1. (B) Nondenaturating PAGE analysis of the cytosine-5 methylation catalyzed by M.CviPI and the successive cleavage of HS2 by GlaI. Lane M, the DNA ladder marker; lane 1, the synthesized HS2; lane 2, the synthesized CP2; lane 3, in the presence of M.CviPI + GlaI + HS2; lane 4, in the presence of GlaI + HS2. (C) Fluorescence measurements of RNase HII-mediated recycling liberation of Cy5 molecules from the AuNP nanostructures in the absence (black line) and presence (red line) of M.SssI. Inset shows the fluorescence intensity of Cy5 in the absence (black column) and presence (red column) of M.SssI. (D) Fluorescence measurements of RNase HII-mediated recycling liberation of Cy3 molecules from the AuNP nanostructures in the absence (grey line) and presence (green line) of M.CviPI. Inset shows the fluorescence intensity of Cy3 in the absence (grey column) and presence (green column) of M.CviPI. The 100 U mL^−1^ M.SssI MTase, 500 U mL^−1^ M.CviPI MTase, 600 nM HS1, 600 nM HS2, 600 nM CP1 and 600 nM CP2 are used in the experiments.

To investigate whether M.SssI and M.CviPI MTases can methylate the cytosine-5 in palindromic sequences 5′-C-G-3′ and 5′-G-C-3′ to initiate RNase HII-mediated recycling liberation of Cy5 and Cy3 molecules from the Cy5/Cy3–signal probe–AuNP nanostructures, respectively, we used nondenaturating polyacrylamide gel electrophoresis (PAGE) and fluorescence spectroscopy to verify the assay feasibility (Fig. 1A). In the presence of GlaI + HS1, only one band of 43 nt is observed (Fig. 1A, lane 4), consistent with the size of the synthesized HS1 (43 nt) (Fig. 1A, lane 1), indicating neither the methylation nor the cleavage of HS1 occurs, while in the presence of M.SssI + GlaI + HS1, two distinct bands of 43 nt and 25 nt are detected (Fig. 1A, lane 3), which are exactly the sizes of the synthesized HS1 (43 nt) (Fig. 1A, lane 1) and CP1 (25 nt) (Fig. 1A, lane 2), indicating that M.SssI MTase can methylate the cytosine-5 in the palindromic sequence 5′-A-C-G-T-3′ to induce the cleavage of HS1 by GlaI for the yielding of CP1 (25 nt). Similarly, in the presence of M.CviPI + GlaI + HS2, two distinct bands of 43 nt and 23 nt are observed (Fig. 1B, lane 3), indicating that M.CviPI MTase enables the methylation of cytosine-5 in the palindromic sequence 5′-G-mC-G-C-3′ to induce the cleavage of HS2 by GlaI for the generation of CP2 (23 nt) (Fig. 1B, lane 2), while in the presence of GlaI + HS2, only the band of 43 nt is detected (Fig. 1B, lane 4), consistent with the synthesized HS2 (43 nt) (Fig. 1B, lane 1), implying that neither methylation nor the cleavage of HS2 occurs due to the absence of M.CviPI MTase. To verify the feasibility of the proposed method, we performed fluorescence measurements (Fig. 1C and D). In the absence of DNA MTases, neither a significant Cy5 fluorescence signal (Fig. 1C, black line) nor a significant Cy3 fluorescence signal (Fig. 1D, grey line) is detected. In contrast, an enhanced Cy5 fluorescence signal with a characteristic emission peak at 668 nm (Fig. 1C, red line) in response to M.SssI and an enhanced Cy3 fluorescence signal with a characteristic emission peak at 568 nm (Fig. 1D, green line) in response to M.CviPI MTases are observed. Notably, extremely low background signals are detected in the absence of DNA MTases (Fig. 1C and D, black and grey lines), and this can be ascribed to the following factors: (1) the novel endonuclease GlaI exhibits high specificity toward 5-mC, efficiently inhibiting the nonspecific cleavage; (2) RNase HII enables specific and efficient excision of a single guanine ribonucleotide, preventing the nonspecific amplification; and (3) AuNPs possess strong quenching capability to efficiently quench the fluorophores (*i.e.*, Cy5 and Cy3). These results (Fig. 1) demonstrate that the proposed method can be used for the simultaneous detection of multiple DNA MTase activities.

**Fig. 2:**
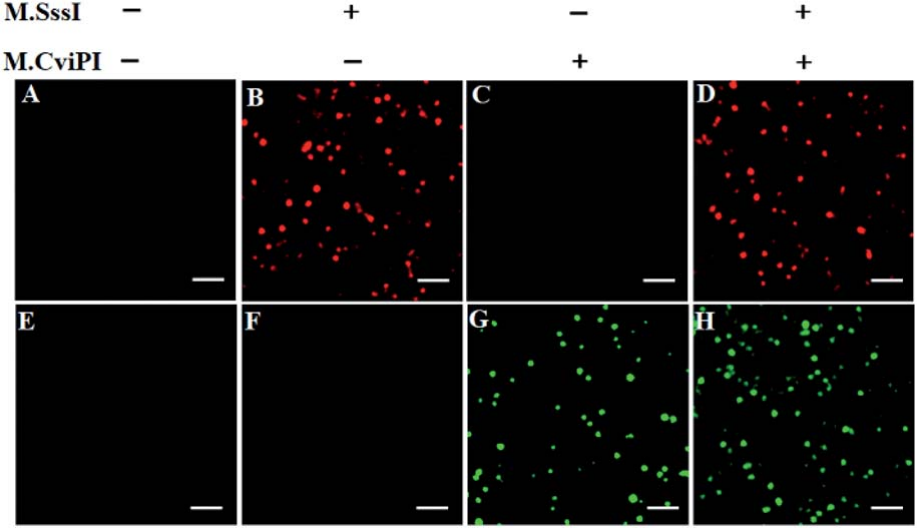
Single-molecule imaging in the absence (A and E) and presence of M.SssI MTase (B and F), M.CviPI MTase (C and G) and M.SssI MTase + M.CviPI MTase (D and H), respectively. The Cy5 fluorescence signals are shown in red color, and the Cy3 fluorescence signals are shown in green color. The 100 U mL^−1^ M.SssI MTase and 500 U mL^−1^ M.CviPI MTase are used in the experiments. The scale bar is 2 μm.

For TIRF-based fluorescence imaging, only fluorescent molecules within 100 nm of the coverslip can be illuminated.^48^ In the Cy5/Cy3–signal probe–AuNP nanostructure, the largest separation distances between AuNP and fluorophores (Cy5 and Cy3) are calculated to be 6.36 nm and 6.02 nm (two adjacent bases for dsDNA are 0.34 nm, and the diameter of the AuNP is 10 nm),^49^ respectively, within the efficient quenching distance (2.2–16.6 nm) of AuNPs.^50^ Because of the largest separation distances (6.36 nm and 6.02 nm) within the excitation field of TIRF (100 nm), the Cy5 and Cy3 molecules can be efficiently imaged by TIRF. Fig. 2 shows the fluorescence images of Cy5 and Cy3 molecules liberated from the Cy5/Cy3–signal probe–AuNP nanostructures as a result of the cytosine-5 methylation-directed RNase HII-mediated recycling cleavage reaction. In the absence of M.SssI and M.CviPI MTases, neither the Cy5 (Fig. 2A) nor Cy3 fluorescence signal (Fig. 2E) is observed, indicating no occurrence of either DNA methylation or the recycling cleavage reaction. In contrast, in the presence of M.SssI MTase, distinct Cy5 fluorescence signals are observed (Fig. 2B, red color), but no Cy3 fluorescence signal is detected (Fig. 2F), indicating that M.SssI can specifically catalyze the cytosine-5 methylation in the palindromic sequence 5′-C-G-3′ and induce the recycling cleavage of SP1 to liberate Cy5 molecules from the Cy5/Cy3–signal probe–AuNP nanostructures. In the presence of M.CviPI MTase, distinct Cy3 fluorescence signals are observed (Fig. 2G, green color), but no Cy5 fluorescence signal is detected (Fig. 2C), indicating that M.CviPI can specifically catalyze the cytosine-5 methylation in the palindromic sequence 5′-G-C-3′ and induce the recycling cleavage of SP2 to liberate Cy3 molecules from the Cy5/Cy3–signal probe–AuNP nanostructures. In the presence of both M.SssI and M.CviPI MTases, distinct Cy5 (Fig. 2D, red color) and Cy3 fluorescence signals (Fig. 2H, green color) are simultaneously observed. These results (Fig. 2) clearly demonstrate that single-molecule detection of Cy5 and Cy3 molecules enables simultaneous measurement of M.SssI and M.CviPI MTase activities.

**Fig. 3:**
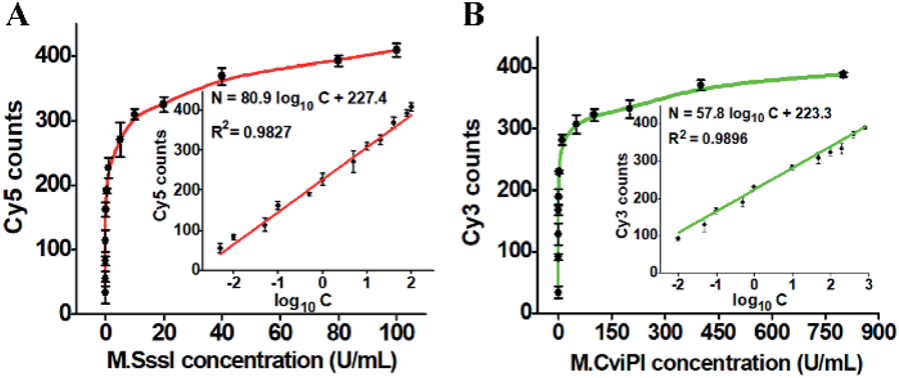
(A) Measurement of Cy5 counts in response to different-concentration M.SssI MTase. The inset shows the linear relationship between the Cy5 counts and the logarithm of M.SssI MTase concentration. (B) Measurement of Cy3 counts in response to different-concentration M.CviPI MTase. The inset shows the linear relationship between Cy3 counts and the logarithm of M.CviPI MTase concentration. The error bars represent the standard deviations of three independent experiments.

Under the optimized experimental conditions (Fig. S2–S5†), we investigated the sensitivity of the proposed method by measuring the fluorescent molecule counts in response to different-concentration DNA MTases. When the M.SssI MTase concentration increases from 0.005 to 100 U mL^−1^, the Cy5 counts increase in a concentration-dependent manner (Fig. 3A). The Cy5 counts exhibit a linear correlation with the logarithm of M.SssI concentration in the range from 0.005 to 100 U mL^−1^. The regression equation is *N* = 80.9 log_10_ *C* + 227.4 with a correlation coefficient of 0.9827, where *N* is the Cy5 counts and *C* is the M.SssI MTase concentration (U mL^−1^) (inset of Fig. 3A). The detection limit is calculated to be 2.01 × 10^−3^ U mL^−1^ by evaluating three times the standard deviation over the signal of negative control without M.SssI MTase. The sensitivity of this method has been improved by more than 497.5-fold compared with that of the colorimetric assay based on enzyme-linkage-mediated AuNP aggregation (1.0 U mL^−1^),^18^ 164.2-fold compared with that of the electrochemical assay based on a photoelectrochemical (PEC) immunosensor (0.33 U mL^−1^),^51^ 49.8-fold compared with that of the luminescence assay based on DNA-templated silver nanoclusters (0.1 U mL^−1^),^52^ and 29.9-fold compared with that of the fluorescence assay based on methylation-sensitive cleavage coupled with the nicking enzyme-assisted signal amplification (0.06 U mL^−1^),^30^ and even is comparable to that of the fluorescence assay based on SDA and DNAzyme amplification (0.0082 U mL^−1^).^53^ As shown in Fig. 3B, the Cy3 counts enhance in a concentration-dependent manner when the M.CviPI MTase concentration increases from 0.01 to 800 U mL^−1^, and the Cy3 counts exhibit a linear correlation with the logarithm of the M.CviPI MTase concentration in the range from 0.01 to 800 U mL^−1^. The regression equation is *N* = 57.8 log_10_ *C* + 223.3 with a correlation coefficient of 0.9896, where *N* is the measured Cy3 counts and *C* is the M.CviPI MTase concentration (U mL^−1^) (inset of Fig. 3B), respectively. The detection limit is calculated to be 3.39 × 10^−3^ U mL^−1^ by evaluating three times the standard deviation over the signal of the negative control without M.CviPl MTase, superior to those of the reported DNA MTase assays.^19–24,26^ The improved sensitivity of the propose method can be ascribed to (1) the good specificity of GlaI toward the methylated DNAs, (2) the high efficiency of the RNase HII-mediated recycling amplification, and (3) the high signal-to-noise ratio of single-molecule detection.

**Fig. 4:**
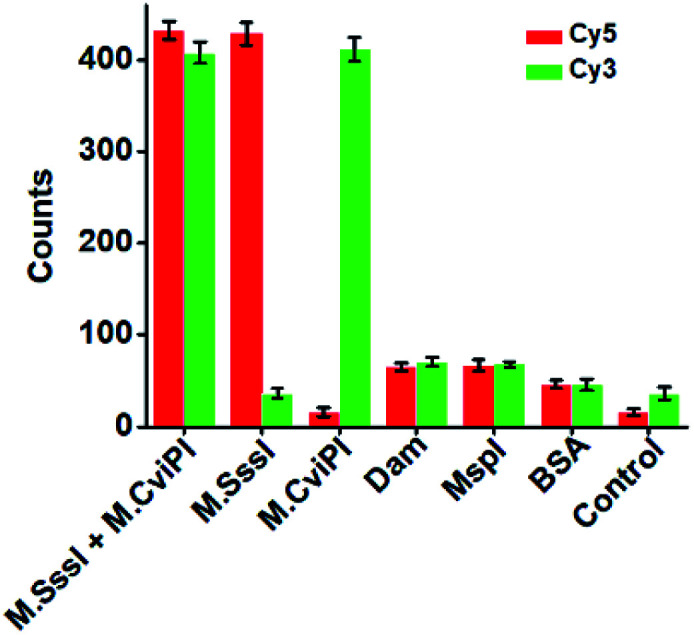
Measurement of Cy5 and Cy3 counts in response to 100 U mL^−1^ M.SssI MTase + 500 U mL^−1^ M.CviPI MTase, 100 U mL^−1^ M.SssI MTase, 500 U mL^−1^ M.CviPI MTase, 100 U mL^−1^ Dam MTase, 100 U mL^−1^ MspI MTase, 0.1 g L^−1^ BSA, and the control with only reaction buffer, respectively. The error bars represent the standard deviations of three independent experiments.

DNA MTase is a superfamily with a large group of members, and it is a great challenge to discriminate one kind of MTase from the others. To investigate the selectivity of the proposed method, we used Dam MTase, MspI MTase, and bovine serum albumin (BSA) as the negative controls. Even though Dam and MspI MTases are the members of the DNA MTase family, they only methylate the N^6^ position of the adenine residue and the C^5^ position of the first cytosine residue in the palindromic sequences 5′-G-A-T-C-3′ and 5′-C-C-G-G-3′, respectively.^19,54^ BSA is not a kind of DNA MTase, and it cannot catalyze the transfer of a methyl group to any base residue in genomic DNA. In the presence of Dam MTase, MspI MTase and BSA, neither the Cy5 nor Cy3 fluorescence signal is observed, consistent with the control with only reaction buffer (Fig. 4). In contrast, in the presence of M.SssI MTase, an enhanced Cy5 fluorescence signal is observed, but no Cy3 fluorescence signal is detected. In the presence of M.CviPI MTase, an enhanced Cy3 fluorescence signal is detected, but no Cy5 fluorescence signal is detected. Moreover, in the presence of both M.SssI and M.CviPI MTases, both Cy5 and Cy3 fluorescence signals can be simultaneously detected (Fig. 4). These results demonstrate that the proposed method exhibits excellent selectivity toward M.SssI and M.CviPI MTases.

**Fig. 5:**
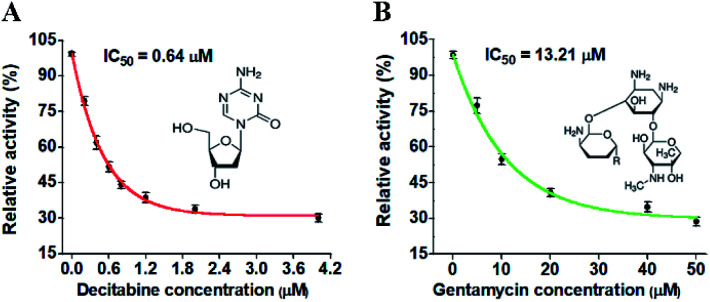
(A) Variance of the relative activity of M.SssI MTase in response to different-concentration decitabine. (B) Variance of the relative activity of M.CviPI MTase in response to different-concentration gentamycin. The 100 U mL^−1^ M.SssI MTase and 500 U mL^−1^ M.CviPI MTase are used in the experiments. The error bars represent the standard deviations of three independent experiments.

DNA MTases are not only important biomarkers but also critical pharmacological targets, and thus the efficient screening of potential inhibitors is essential to clinical diagnosis and cancer therapy. To investigate the feasibility of the proposed method for the inhibition assay, we used decitabine and gentamycin as the model inhibitors. Decitabine (*i.e.*, 5-aza-2′-deoxycytidine) is a 2′-deoxycytidine analogue, and it can be phosphorylated to form the aza-dCTP.^55^ The aza-dCTP may replace the cytosine in CpG islands to inhibit DNA methylation through forming a covalent bond with the DNA MTase enzyme.^55^ Gentamycin is a kind of aminoglycoside antibiotic with a broad-spectrum antimicrobial activity, and it has been widely used as a methyl transferase inhibitor.^56^ When the decitabine concentration increases from 0.2 to 4 μM, the relative activity of M.SssI MTase decreases in a concentration-dependent manner (Fig. 5A). The IC_50_ value (half-maximal inhibitory concentration) is calculated to be 0.64 μM according to the fitted calibration curve (Fig. 5A), consistent with the value (0.50 μM) obtained by the fluorescent assay based on SDA and DNAzyme amplification^53^ and the value (0.63 μM) obtained by the fluorescent assay based on graphene oxide.^57^ Similarly, when the gentamycin concentration increases from 5 to 50 μM, the relative activity of M.CviPI MTase decreases correspondingly (Fig. 5B), and the IC_50_ value is calculated to be 13.21 μM according to the fitted calibration curve (Fig. 5B). These results clearly demonstrate that the proposed method can be used for the simultaneous screening of multiple DNA MTase inhibitors.

**Fig. 6:**
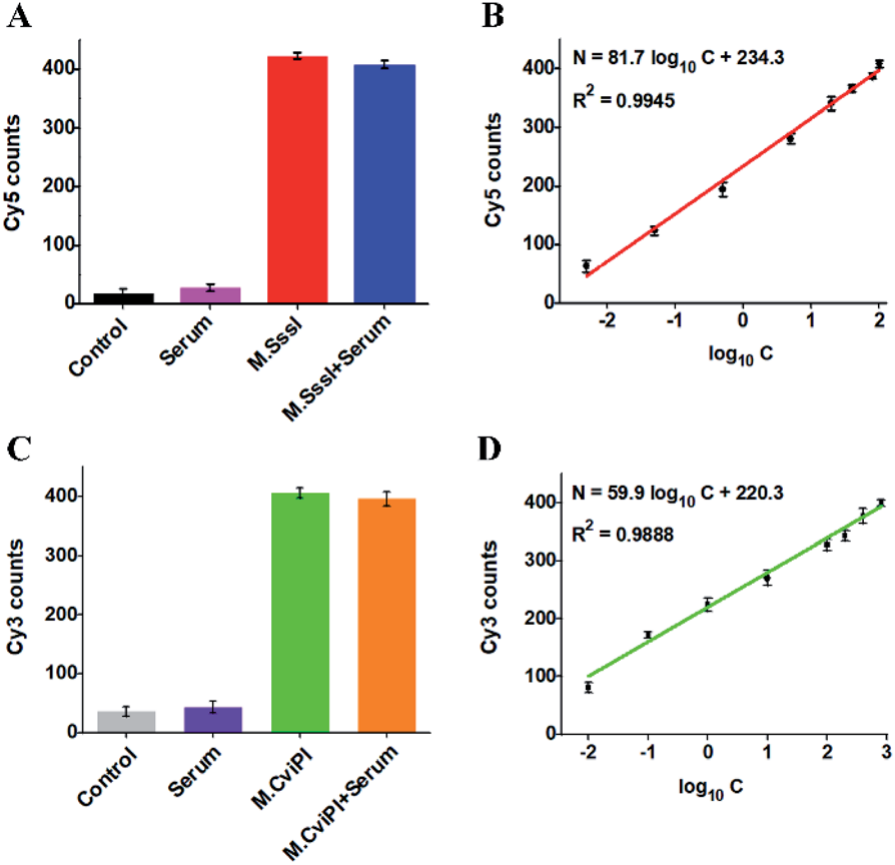
(A) Variance of Cy5 counts in response to the control without both M.SssI MTase and human serum (black column), 10% human serum (pink column), 100 U mL^−1^ M.SssI MTase (red column), and 100 U mL^−1^ M.SssI MTase + 10% human serum (blue column), respectively. (B) Linear relationship between Cy5 counts and the logarithm of M.SssI MTase concentration in 10% human serum. (C) Variance of Cy3 counts in response to the control without M.CviPI MTase and human serum (grey column), 10% human serum (purple column), 500 U mL^−1^ M.CviPI MTase (green column), and 500 U mL^−1^ M.CviPI MTase + 10% human serum (orange column), respectively. (D) Linear relationship between Cy3 counts and the logarithm of M.CviPI MTase concentration in 10% human serum. The error bars represent the standard deviations of three independent experiments.

The accurate and sensitive detection of multiple DNA MTases in real samples is of great significance to biomedical research and clinical diagnosis. To verify the feasibility of the proposed method for real sample analysis, we measured the fluorescent molecule counts in response to DNA MTases spiked in 10% normal human serum (Fig. 6). In the absence of M.SssI MTase, no distinct Cy5 signal is observed in either the control without human serum (Fig. 6A, black column) or only human serum (Fig. 6A, pink column). In contrast, an enhanced Cy5 signal is detected in response to M.SssI MTase (Fig. 6A, red column), consistent with the value obtained in the presence of M.SssI MTase + 10% human serum (Fig. 6A, blue column). Notably, the Cy5 counts enhance with the increasing concentration of M.SssI MTase in human serum, and the Cy5 counts exhibit a linear correlation with the logarithm of M.SssI MTase concentration in the range from 0.005 to 100 U mL^−1^ (Fig. 6B). The regression equation is *N* = 81.7 log_10_ *C* + 234.3 with a correlation coefficient of 0.9945, where *N* is the Cy5 counts and *C* is the M.SssI MTase concentration (U mL^−1^). The detection limit is calculated to be 2.47 × 10^−3^ U mL^−1^ based on the evaluation of three times the standard deviation plus the average signal of blank, comparable to the value (2.01 × 10^−3^ U mL^−1^) obtained in the absence of 10% human serum (Fig. 3A). As shown in Fig. 6C, no distinct Cy3 signal is observed in either the control without human serum (Fig. 6C, grey column) or only human serum (Fig. 6C, purple column). In contrast, an enhanced Cy3 signal is detected in the presence of M.CviPI MTase (Fig. 6C, green column), comparable to the value obtained in the presence of M.CviPI MTase + 10% human serum (Fig. 6C, orange column). With the increasing concentration of M.CviPI MTase in human serum, the Cy3 counts exhibit a linear correlation with the logarithm of M.CviPI MTase concentration in the range from 0.01 to 800 U mL^−1^ (Fig. 6D). The regression equation is *N* = 59.9 log_10_ *C* + 220.3 with a correlation coefficient of 0.9888, where *N* is the Cy3 counts and *C* is the M.CviPI MTase concentration (U mL^−1^). The detection limit is calculated to be 4.57 × 10^−3^ U mL^−1^ based on the evaluation of three times the standard deviation plus the average signal of blank, comparable to the value (3.39 × 10^−3^ U mL^−1^) obtained in the absence of human serum (Fig. 3B). Furthermore, we evaluated the recovery ratios of DNA MTases by spiking different-concentration M.SssI MTase (1–40 U mL^−1^) and M.CviPI MTase (10–500 U mL^−1^) in normal human serum. As shown in Tables S1 and S2,† the recovery ratios are calculated to be 97.38–107.68% with a relative standard deviation (RSD) of 1.37–2.08% for M.SssI MTase and 97.96–103.65% with a RSD of 0.85–2.82% for M.CviPI MTase, consistent with the values (recovery ratio of 99.8–109.0% with a RSD of 2.50–4.10%) obtained by the fluorescence assay based on SDA and DNAzyme amplification,^53^ and the values (recovery ratio of 91.7–98.5% with a RSD of 3.84–7.13%) obtained by the fluorescence assay based on methylation-sensitive cleavage coupled with nicking enzyme-assisted signal amplification.^30^ Moreover, this method exhibits good performance in human embryonic kidney cell (HEK-293 cells) lysate samples.^58^ When 2.5 μg of diluted HEK-293 cell lysate samples are spiked with M.SssI and M.CviPI MTases, respectively, the measured M.SssI and M.CviPI activities (Fig. S6†) are consistent with the values obtained in the absence of 10% human serum (Fig. 3), and the recovery ratios (Tables S3 and S4†) are comparable to the values obtained in the samples spiked with human serum (Tables S1 and S2†). These results demonstrate that the proposed method exhibits good tolerance toward interferents from the normal human serum and cell lysates, and it can accurately detect multiple DNA MTase activities in complex real samples.

## Conclusions

In summary, we demonstrate for the first time the cytosine-5 methylation-directed construction of a AuNP-based nanosensor for simultaneous detection of multiple DNA MTases at the single-molecule level. In this assay, we introduce a novel methyl-directed endonuclease GlaI for cleaving site-specific 5-mC and a single-molecule detection technique for accurately counting the released Cy3 and Cy5 molecules. In addition, we ingeniously design two hairpin substrates containing the dinucleotide sequence 5′-A-C-G-T-3′ for CpG MTase (*i.e.*, M.SssI MTase), and the dinucleotide sequence 5′-G-mC-G-C-3′ for GpC MTase (*i.e.*, M.CviPI MTase), which can be methylated at cytosine-5 to form the catalytic substrates for GlaI. The cleavage of catalytic substrates by GlaI may induce the recycling liberation of large numbers of Cy5 and Cy3 molecules from the Cy5/Cy3–signal probe–AuNP nanostructures, enabling simultaneous detection of multiple DNA MTases. Due to the good specificity of GlaI toward methylated DNAs, the high efficiency of the RNase HII-mediated recycling amplification, and the high signal-to-noise ratio of single-molecule detection, the proposed method exhibits good specificity and high sensitivity with a detection limit of 2.01 × 10^−3^ U mL^−1^ for M.SssI MTase and 3.39 × 10^−3^ U mL^−1^ for M.CviPI MTase over a large dynamic range of 4 orders of magnitude, superior to most of the reported DNA MTase assays.^18,30,51–53^ Moreover, this method can be applied to discriminate different DNA MTases, screen the DNA MTase inhibitors, and measure DNA MTase activities in human serum and cell lysate samples. Importantly, this method may be extended to simultaneous detection of other CpG and GpC MTases by rationally designing appropriate DNA substrates, holding great potential in biomedical research, clinical diagnosis, drug discovery and cancer therapeutics.

## Experimental section

### Chemicals and materials

All the oligonucleotides (Table 1) were synthesized at Shanghai Sangon Biological Engineering Technology & Services Co. Ltd. (Shanghai, China). CpG methyltransferase (M.SssI MTase), 10× NEBuffer 2 (500 mM sodium chloride (NaCl), 100 mM trizma hydrochloride (Tris–HCl), 100 mM magnesium chloride (MgCl_2_), 10 mM dl-dithiothreitol (DTT), pH 7.9), GpC methyltransferase (M.CviPI MTase), 10× GC reaction buffer (500 mM NaCl, 500 mM Tris–HCl, 100 mM DTT, pH 8.5), ribonuclease HII (RNase HII), 10× ThermoPol reaction buffer pack (200 mM Tris–HCl, 100 mM potassium chloride (KCl), 100 mM ammonium sulfate ((NH_4_)_2_SO_4_), 20 mM magnesium sulfate (MgSO_4_), 1% Triton X-100, pH 8.8), DNA adenine methyltransferase (Dam MTase), MspI MTase, and *S*-adenosylmethionine (SAM) were purchased from New England Biolabs (Ipswich, MA, USA). Methyl-directed DNA endonuclease (GlaI) and 10× SEBuffer Y (330 mM Tris-acetate (Tris-Ac), 660 mM potassium acetate (KAc), and 100 mM magnesium acetate (Mg(Ac)_2_), 10 mM DTT, pH 7.9) were obtained from SibEnzyme Ltd. (Ak, Novosibirsk, Russia). Decitabine, gentamycin, and bovine serum albumin (BSA) were purchased from Sigma Aldrich Co (St. Louis, MO, USA). SYBR Gold was obtained from Life Technologies (Carlsbad, CA, USA). Au nanoparticles (AuNPs) were bought from Nanocs Inc. (New York, NY, USA). Ultrapure water was prepared by a Millipore filtration system (Millipore, Milford, MA, USA).

**Table 1:** Sequences of the oligonucleotides^a^

Note	Sequences (5′–3′)
HS1	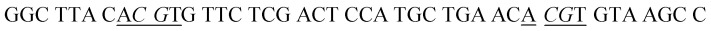
HS2	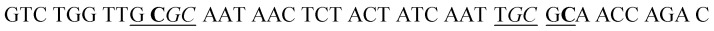
SP1	AGC AT̲*G* GAG TCG ATT–SH
SP2	TGA T̲A*G* TAG AGT TTT–SH
CP1	GTG TTC TCG ACT CCA TGC TGA ACA C
CP2	GCA ATA ACT CTA CTA TCA ATT GC

a In HS1 and HS2, the underlined regions signify the recognition sites for GlaI, and the italicized regions signify the recognition sites for M.SssI and M.CviPI MTases, respectively. In HS2, the bold base “C” indicates the modified 5-mC. In SP1 and SP2, the underlined bases “T” indicate the modification with Cy5 and Cy3, respectively, and the italicized bases “G” indicate guanine ribonucleotides, and the “SH” indicates the modification of a sulfhydryl group.

### Preparation of signal probe-functionalized AuNPs

The AuNPs (10 nm) were functionalized with fluorophores (Cy5 and Cy3), and the SH-modified signal probes were prepared through a salt aging method.^59^ Both signal probe 1 (SP1, 6.6 nmol) and signal probe 2 (SP2, 6.8 nmol) were added into the AuNP solution (1 mL, 5.7 × 10^12^ particles per mL), followed by standing at room temperature in PBS buffer (10 mM phosphate (NaH_2_PO_4_/Na_2_HPO_4_), pH 7.4). After standing for 20 min, NaCl (2 M NaCl dissolved in 10 mM PBS buffer) was added into the above solution with a final concentration of 0.02 M. The obtained DNA–AuNP suspension was sonicated for 20 s, followed by incubation at room temperature for 20 min. This process was repeated for every 0.1 M NaCl increment until reaching a final concentration of 0.5 M. The salting process was followed by overnight incubation at room temperature. To remove the excess signal probes, the DNA–AuNP suspension was centrifuged at 13 000 rpm for 25 min, and the supernatants were removed. The resultant DNA–AuNPs were resuspended in PBS buffer (60 μL, 10 mM phosphate, 0.1 M NaCl, pH 7.0) and stored at 4 °C for further use. In the signal probe-modified AuNP solution, the concentration of the signal probe was estimated to be 22.1 μM (Fig. S1†).

### Cytosine-5 methylation-directed construction of a AuNP-based nanosensor for simultaneous detection of multiplex DNA MTases

The DNA MTase assay involves three consecutive steps. First, all oligonucleotides were diluted with 1× Tris–EDTA buffer (10 mM Tris, 1 mM EDTA, pH 8.0) to prepare a stock solution. Hairpin substrate 1 (HS1) and hairpin substrate 2 (HS2) were diluted to 10 μM in a hybridization buffer (10 mM Tris–HCl, 1.5 mM MgCl_2_, pH 8.0) and incubated at 95 °C for 5 min, followed by slowly cooling to room temperature for the perfect formation of the hairpin structure. 1.2 μL of resultant HS1 and HS2 was added into 20 μL of reaction solution (different-concentration M.SssI and M.CviPI MTases, 320 μM SAM, 2 μL of 10× NEBuffer 2, and 2 μL of 10× GC reaction buffer), and incubated at 37 °C for 2 h. Second, 10 μL of methylation products was added into 10 μL of reaction solution (2 U of GlaI, and 2 μL of 10× SEBuffer Y), and incubated at 30 °C for 80 min. Third, 10 μL of excision products were added into 20 μL of reaction solution (4 μL of SP1/SP2–AuNP, 5 U of RNase HII, 3 μL of 10× ThermoPol reaction buffer pack, and 3 μL of 10× hybridization buffer (10 mM Tris–HCl, 50 mM NaCl and 1 mM EDTA (pH 8.0))), and incubated at 37 °C for 40 min to carry out the RNase HII-mediated recycling cleavage reaction.

### Fluorescence measurements and electrophoresis analysis

The 30 μL of amplification products was diluted to 60 μL with ultrapure water. The fluorescence spectra were measured with a HitachiF-7000 fluorescence spectrophotometer (Tokyo, Japan). The emission spectra were recorded at a scan rate of 2 nm s^−1^ with an excitation wavelength of 640 nm for Cy5 and 540 nm for Cy3, and the fluorescence intensities at the emission wavelength of 668 nm for Cy5 and 568 nm for Cy3 were recorded for data analysis, respectively. In order to analyze the cleavage products of GlaI, 12% nondenaturating polyacrylamide gel electrophoresis (PAGE) was performed in 1× TBE buffer (9 mM Tris–HCl, 0.2 mM EDTA, and 9 mM boric acid, pH 7.9) at a constant voltage of 110 V for 45 min at room temperature. After electrophoresis, the gels were stained with SYBR Gold, and visualized with a ChemiDoc MP Imaging system (Hercules, California, USA).

### Inhibition assay

For the M.SssI MTase inhibition assay, different-concentration decitabine was added into the reaction mixture (600 nM HS1, 100 U mL^−1^ M.SssI, 320 μM SAM, and 2 μL of 10× NEBuffer 2), and incubated at 37 °C for 2 h. For the M.CviPI MTase inhibition assay, different-concentration gentamycin was added into the reaction mixture (600 nM HS2, 500 U mL^−1^ M.CviPI, 320 μM SAM, and 2 μL of 10× GC reaction buffer), and incubated at 37 °C for 2 h. Subsequently, the reaction products were used for the detection of M.SssI and M.CviPI MTase activities, respectively, according to the procedures described above. The relative activities (RA) of M.SssI and M.CviPI MTases were calculated based on eqn (1).

1 RA (%) = (*N*_i_ − *N*_0_)/(*N*_t_ − *N*_0_) × 100%

where *N*_0_ is the fluorescent molecule (Cy5/Cy3) counts in the absence of M.SssI MTase/M.CviPI MTase, respectively; *N*_t_ is the fluorescent molecule (Cy5/Cy3) counts in the presence of 100 U mL^−1^ M.SssI MTase/500 U mL^−1^ M.CviPI MTase, respectively; *N*_i_ is the fluorescent molecule (Cy5/Cy3) counts in the presence of M.SssI MTase + decitabine/M.CviPI MTase + gentamycin, respectively.

### Single-molecule imaging and data analysis

The reaction products were diluted 300-fold with the imaging buffer (0.4% (w/v) d-glucose, 1 mg mL^−1^ glucose oxidase, 50 μg mL^−1^ BSA, 67 mM glycine–KOH, 0.04% mg mL^−1^ catalase, 2.5 mM MgCl_2_, and 1 mg mL^−1^ trolox, pH 9.4). Then 10 μL of the diluted samples were spread on a glass coverslip (20 × 40 mm, Menzel-Glaser, Braunschweig, Germany) for TIRF-based single-molecule imaging. The Cy5 and Cy3 molecules were excited by the sapphire 640 and 561 nm lasers (Coherent) simultaneously, and the resulting photons were collected by an oil immersion objective (CFI Apochromat TIRF 100×). The Cy5 and Cy3 fluorescence signals were split up into two channels of 661.5–690.5 nm and 573–613 nm filters, and imaged on an Andor ixon Ultra 897 EMCCD camera. For data analysis, the image J software was used for counting the fluorescent molecules (Cy5 and Cy3). The resulting numbers of fluorescent molecules (Cy5 and Cy3) are the average values of five images, respectively.

### Simultaneous detection of multiple DNA MTases in real samples

For cell lysate preparation, HEK-293 cells were cultured in 5% CO_2_ at 37 °C in Dulbecco's modified Eagle's medium containing 10% fetal bovine serum and 1% penicillin–streptomycin. The cells were collected and pelleted at 1200 rpm at 4 °C, followed by the addition of 100 μL of lysis buffer (25 mM Tris–HCl, pH 7.6, 150 mM NaCl, 1% NP-40, 1% sodium deoxycholate, 1% SDS) supplemented with 1× protease inhibitor cocktail, 1 mM phenylmethylsulfonyl fluoride, and 1 mM activated sodium orthovanadate. Subsequently, the cells were incubated on ice for 10 min, and centrifuged at 12 000 rpm at 4 °C for 15 min. The total protein concentration was determined using a BCA protein assay kit (Pierce, Rockford, USA). The obtained lysates were diluted to a final amount of 2.5 μg with a PBS buffer, and spiked with DNA MTases in the reaction mixture for the MTase activity assay. For recovery ratio analysis, different-concentration M.SssI MTase was spiked into the reaction mixtures containing 10% human serum (or HEK-293 cell lysates), 600 nM HS1, 320 μM SAM, and 2 μL of 10× NEBuffer 2, and incubated at 37 °C for 2 h. Similarly, different-concentration M.CviPI MTase was spiked into the reaction mixture containing 10% human serum (or HEK-293 cell lysates), 600 nM HS2, 320 μM SAM, and 2 μL of 10× GC reaction buffer, and incubated at 37 °C for 2 h. The M.SssI and M.CviPI MTase activities were measured using the procedures described above, and determined according to the fitting equations (inset of Fig. 3A and B). The recovery ratios (*R*) were calculated based on eqn (2).

2 *R* (%) = (*C*_m_/*C*_o_) × 100%

where *C*_m_ is the concentration of M.SssI and M.CviPI MTases in the presence of human serum (or HEK-293 cell lysates), respectively, and *C*_0_ is the concentration of M.SssI and M.CviPI MTases in the absence of human serum (or HEK-293 cell lysates), respectively.

## Conflicts of interest

There are no conflicts to declare.

## Supplementary Material

SC-011-D0SC03240A-s001
