# Supplementary material for: Cytosine-5 methylation-directed construction of a Au nanoparticle-based nanosensor for simultaneous detection of multiple DNA methyltransferases at the single-molecule level
Source: Chem Sci. 2020 Aug 25;11(35):9675–84. doi: 10.1039/d0sc03240a (PMC8161687; doi:10.1039/d0sc03240a)
Supplement: SC-011-D0SC03240A-s001 [file SC-011-D0SC03240A-s001.pdf]

Electronic Supplementary Material (ESI) for Chemical Science.
This journal is © The Royal Society of Chemistry 2020

Electronic Supplementary Information

Cytosine-5 methylation-directed construction of Au nanoparticle-based nanosensor for simultaneous detection of multiple DNA methyltransferases at the single-molecule level

Li-juan Wang,[†,a] Xiao Han,[†,a] Jian-Ge Qiu, [b] BingHua Jiang*[,b] and Chun-yang Zhang*[,a]

[a] College of Chemistry, Chemical Engineering and Materials Science, Collaborative Innovation Center of Functionalized Probes for Chemical Imaging in Universities of Shandong, Key Laboratory of Molecular and Nano Probes, Ministry of Education, Shandong Provincial Key Laboratory of Clean Production of Fine Chemicals, Shandong Normal University, Jinan 250014, China.

[b] Academy of Medical Sciences, Zhengzhou University, Zhengzhou 450000, China.

* Corresponding author. Tel.: +86 0531-86186033; Fax: +86 0531-82615258. E-mail: cyzhang@sdnu.edu.cn, bhjiang@zzu.edu.cn.

[†] These authors contributed equally.

1. Characterization of Cy5/Cy3-SP1/SP2-functioned AuNPs and the number of signal probes per AuNP.

To verify the signal probes bound to the surface of AuNPs through the covalent S-Au bond, we measured the UV absorption spectra of the signal probe-functioned AuNPs. As shown in Fig. S1, only an absorption peak at 520 nm is detected in the presence of AuNPs (black curve), and the absorption peaks at 260 nm, 670 nm (pink curve) and 570 nm (blue curve) are observed in the presence of Cy5-SP1 and Cy3-SP2, respectively. In contrast, two absorption peaks at 260 nm and 520 nm are detected in the presence of Cy5/Cy3-SP1/SP2-functioned AuNPs (red curve). These results demonstrate that the signal probes are successfully bound to the AuNPs. The number of signal probes per AuNP is calculated by measuring AuNPs and signal probes, respectively. Because 1 mL of AuNPs (5.7×10^{12} particles/mL) is used to bind the signal probes and the concentration of signal probes functioned on the AuNP surface is determined to be 22.1 μM in the final volume of 60 μL, the number of signal probes per AuNP is estimated to be 140.

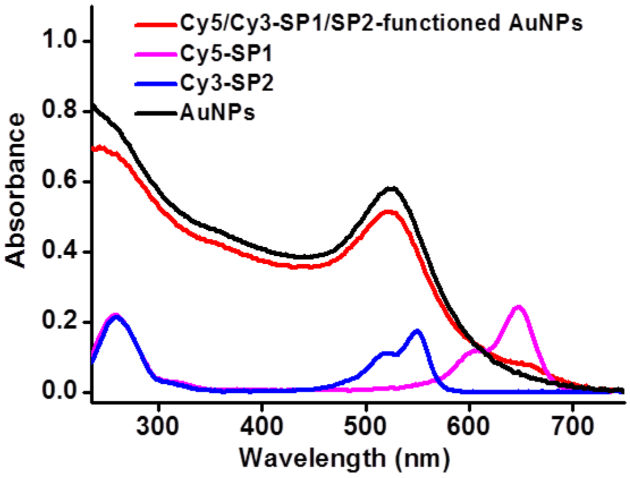

Fig. S1 UV-Vis absorption spectra of AuNPs (black color), Cy5-SP1 (10 μM, pink color), Cy3-SP2 (10 μM, blue color), and Cy5/Cy3-signal probe-functioned AuNPs (red color).

2. Optimization of the amount of DNA endonuclease GlaI.

DNA endonuclease GlaI can cleave the methylated hairpin substrates to yield the capture probes, inducing subsequent RNase HII-mediated recycling cleavage of signal probes to release Cy5 and Cy3 molecules from the Cy5/Cy3-SP1/SP2-AuNP nanostructures. Thus, the amount of GlaI should be optimized. As shown in Fig. S2, the fluorescence intensity enhances gradually with the increasing amount of GlaI from 1 to 5 U, and reaches the plateau beyond the amount of 4 U. Therefore, 4 U of GlaI is used in the subsequent research.

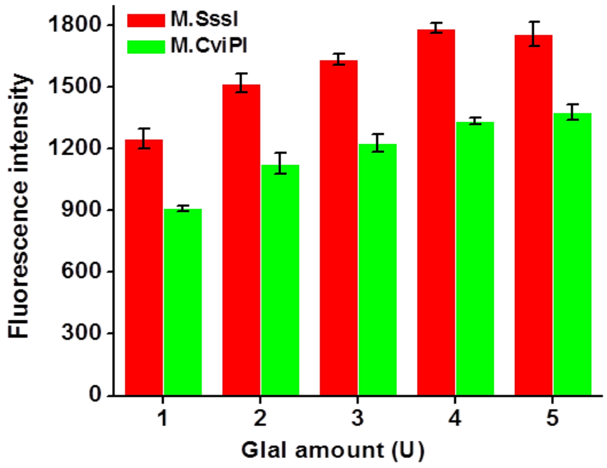

Fig. S2 Variance of fluorescence intensity with different amounts of GlaI in the range from 1 to 5 U. The error bars represent standard deviations of three independent experiments.

3. Optimization of reaction time of GlaI.

The reaction time of GlaI affects the cleavage efficiency of hairpin substrates, which has close association with the amplification efficiency of RNase HII-mediated recycling cleavage reaction. Thus, the reaction time of GlaI should be optimized. As shown in Fig. S3, the fluorescence intensity enhances with reaction time from 10 to 80 min, and reaches the maximum value at 80 min. However, the fluorescence intensity decreases beyond 80 min, because long reaction time

may induce the nonspecific cleavage of hairpin substrates to yield nonspecific capture probes. Thus, 80 min is used as the optimal reaction time of GlaI.

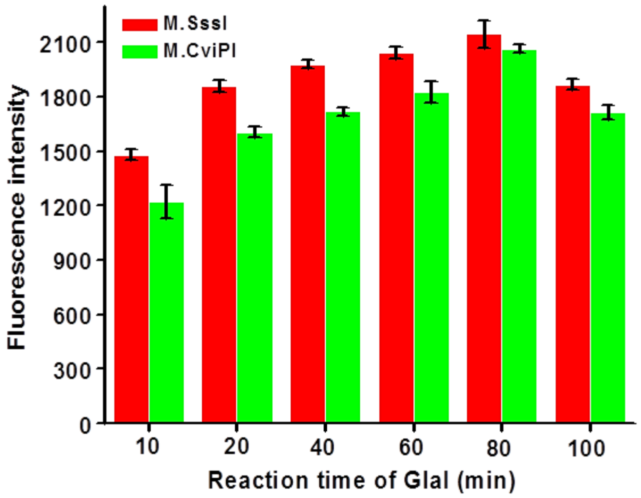

Fig. S3 Variance of fluorescence intensity with reaction time of GlaI in the range from 10 to 100 min. The error bars represent standard deviations of three independent experiments.

4. Optimization of the amount of RNase HII.

RNase HII is responsible for the recycling cleavage of signal probes to liberate Cy5 and Cy3 molecules from the Cy5/Cy3-SP1/SP2-AuNP nanostructures, and it should be optimized. As shown in Fig. S4, the fluorescence intensity enhances with the increasing amount of RNase HII from 2 to 6 U, and reaches the plateau beyond the amount of 5 U. Thus, 5 U is used as the optimal amount of RNase HII.

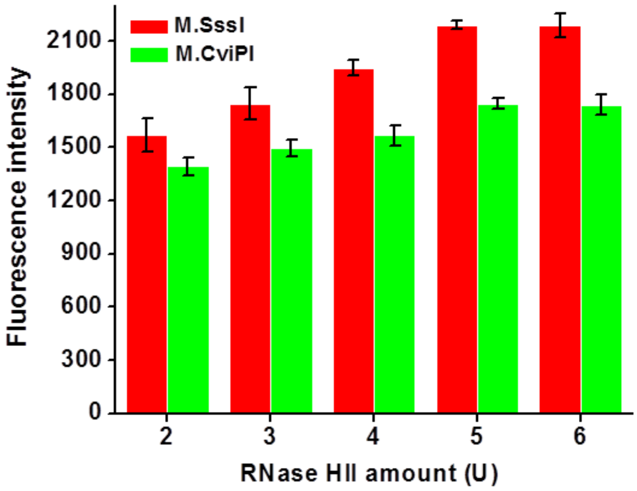

Fig. S4 Variance of fluorescence intensity with different amounts of RNase HII in the range from 2 to 6 U. The error bars represent standard deviations of three independent experiments.

5. Optimization of the reaction time of RNase HII.

The reaction time of RNase HII directly influences the amplification efficiency of the RNase HII-mediated recycling cleavage reaction. To achieve high sensitivity, the optimization of RNase HII reaction time is necessary. As shown in Fig. S5, the fluorescence intensity increases with the reaction time from 10 to 100 min, and reaches the maximum value at 80 min, followed by the decrease beyond 80 min because longer reaction time may induce the nonspecific excision of single guanine ribonucleotide, which is unable to liberate Cy5 and Cy3 molecules from the Cy5/Cy3-SP1/SP2-AuNP nanostructures. Thus, the optimal reaction time of RNase HII is determined to be 40 min.

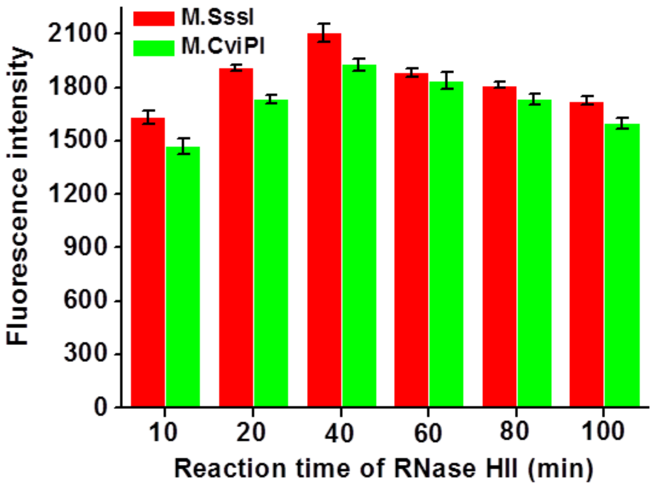

Fig. S5 Variance of fluorescence intensity with reaction time of RNase HII in the range from 10 to 100 min. The error bars represent standard deviations of three independent experiments.

6. Simultaneous detection of multiple DNA MTases in the spiked human serum.

To investigate the feasibility of the proposed method for real sample analysis, we measured the recovery ratios of multiple DNA MTases by spiking different concentrations of M.SssI (1 – 40 U/mL) and M.CviPI (10 – 500 U/mL) MTases into 10% human serum. As shown in Tables S1 and S2, the recovery ratios are calculated to 97.38% – 107.68% with a relative standard deviation (RSD) of 1.37% – 2.08% for M.SssI MTase, and 97.96% – 103.65% with a RSD of 0.85% – 2.82% for M.CviPI MTase, consistent with the values (recovery ratio of 99.8% – 109.0% with a RSD of 2.50% – 4.10%) obtained by the fluorescent assay based on SDA and DNAzyme amplification,[1] and the values (recovery ratio of 91.7% – 98.5% with a RSD of 3.84% – 7.13%) obtained by the fluorescent assay based on methylation-sensitive cleavage coupled with nicking enzyme-assisted signal amplification.[2] These results demonstrate that the proposed method can be applied for simultaneous detection of multiple DNA MTases in human serum, holding great potential in biomedical research and clinical diagnosis.

Table S1. Recovery study by spiking M.SssI MTase into 10% human serum.

sample	added (U/mL)	measured (U/mL)	recovery (%)	RSD (%)
1	1.0	1.08	107.68	1.37
2	5.0	4.87	97.38	2.08
3	10.0	10.51	105.05	1.79
4	20.0	19.65	98.25	2.01
5	40.0	41.21	103.01	1.97

Table S2. Recovery study by spiking M.CviPI MTase into 10% human serum.

sample	added (U/mL)	measured (U/mL)	recovery (%)	RSD (%)
1	10.0	10.37	103.65	1.19
2	50.0	48.98	97.96	2.82
3	100.0	100.39	100.39	1.33
4	200.0	197.61	98.81	2.08
5	500.0	512.86	102.57	0.85

7. Simultaneous detection of multiple DNA MTases in the spiked human cell lysate.

To further verify the feasibility of this proposed method for complex biological samples analysis, we measured the activities and the recovery ratios of M.SssI and M.CviPI MTases in human embryonic kidney cell (HEK-293 cells) lysate samples.[3] Fig. S6A shows the variance of Cy5 counts in response to different-concentration M.SssI MTase spiked in HEK-293 cell lysates. The Cy5 counts improve with the increasing concentration of M.SssI MTase in HEK-293 cell lysates,

and a linear correlation is obtained between the Cy5 counts and the logarithm of M.SssI MTase concentration in the range from 0.005 to 100 U/mL (inset of Fig. S6A). The correlation equation is $N = 76.5 \log_{10} C + 216.9$ with a correlation coefficient of 0.9890, where N is the Cy5 counts and C is the M.SssI MTase concentration (U/mL). The detection limit is calculated to be 3.01×10^{-3} U/mL based on the evaluation of three times the standard deviation plus the average signal of blank, consistent with the value (2.01×10^{-3} U/mL) obtained in the absence of 10% human serum (Fig. 3A). As shown in Fig. S6B, the Cy3 counts enhance with increasing concentration of M.CviPI MTase in HEK-293 cell lysates, and a linear correlation is obtained between the Cy3 counts and the logarithm of M.CviPI MTase concentration in the range from 0.01 to 800 U/mL (inset of Fig. S6B). The correlation equation is $N = 63.3 \log_{10} C + 193.4$ with a correlation coefficient of 0.9855, where N is the Cy3 counts and C is the M.CviPI MTase concentration (U/mL). The detection limit is calculated to be 4.78×10^{-3} U/mL, consistent with the value (3.39×10^{-3} U/mL) obtained in the absence of 10% human serum (Fig. 3B). Moreover, we measured the recovery ratios of DNA MTases by spiking different concentrations of M.SssI MTase (1 – 40 U/mL) and M.CviPI MTase (10 – 500 U/mL) into HEK-293 cell lysates, respectively. As shown in Tables S3 and S4, the measured recovery ratios are 93.20% – 114.20% with a relative standard deviation (RSD) of 1.37% – 2.49% for M.SssI MTase, and 99.37% – 105.56% with a RSD of 1.16% – 2.27% for M.CviPI MTase, consistent with the values obtained in the samples spiked with human serum (Tables S1 and S2). These results demonstrate that the proposed method can be applied for accurate quantification of multiple DNA MTases in complex biological samples.

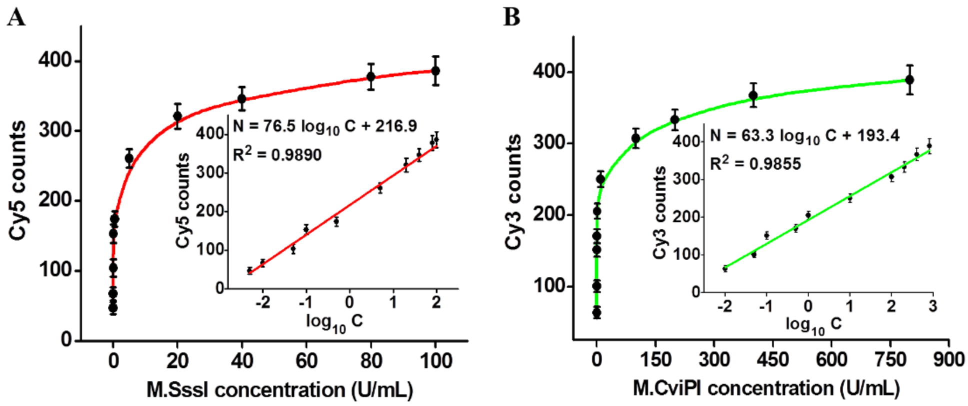

Fig. S6 (A) Measurement of Cy5 counts in response to different-concentration M.SssI MTase in HEK-293 cell lysates. The inset shows the linear relationship between the Cy5 counts and the logarithm of M.SssI MTase concentration in HEK-293 cell lysates. (B) Measurement of Cy3 counts in response to different-concentration M.CviPI MTase in HEK-293 cell lysates. The inset shows the linear relationship between Cy3 counts and the logarithm of M.CviPI MTase concentration in HEK-293 cell lysates. The error bars represent the standard deviations of three independent experiments.

Table S3. Recovery studies by spiking M.SssI MTase into HEK-293 cell lysates.

sample	added (U/mL)	measured (U/mL)	recovery (%)	RSD (%)
1	1.0	1.03	103.0	1.37
2	5.0	4.83	96.60	1.49
3	10.0	11.42	114.20	2.49
4	20.0	18.64	93.20	1.68
5	40.0	42.33	105.83	2.37

Table S4. Recovery studies by spiking M.CviPI MTase into HEK-293 cell lysates.

sample	added (U/mL)	measured (U/mL)	recovery (%)	RSD (%)
1	10.0	10.14	101.40	1.65
2	50.0	49.87	99.74	1.65
3	100.0	105.56	105.56	2.27
4	200.0	198.73	99.37	1.64
5	500.0	506.90	101.38	1.16

References

1. W. Cui, L. Wang and W. Jiang, *Biosens. Bioelectron.*, 2016, **77**, 650-655.
2. Y. Zhao, F. Chen, Y. Wu, Y. Dong and C. Fan, *Biosens. Bioelectron.*, 2013, **42**, 56-61.
3. J. Huang, X. Y. Li, Y. C. Du, L. N. Zhang, K. K. Liu, L. N. Zhu and D. M. Kong, *Biosens. Bioelectron*., 2017, **91**, 417-423.
